# Case Report: A rare case of community-acquired *Roseomonas mucosa* sepsis that presented with persistently normal host-response biomarkers

**DOI:** 10.3389/fimmu.2025.1521161

**Published:** 2025-04-10

**Authors:** Xiaomei Xu, Ying Li, Xin Huang

**Affiliations:** ^1^ Department of Infectious Diseases, Chengdu Fifth People’s Hospital, Chengdu, China; ^2^ Department of Medical Laboratory Technology, Chengdu Fifth People’s Hospital, Chengdu, China

**Keywords:** *Roseomonas mucosa*, sepsis, inflammatory biomarkers, metagenomic next-generation sequencing, blood culture

## Abstract

Community-acquired *Roseomonas mucosa* sepsis can lead to significant morbidity and mortality if not diagnosed promptly. We report a case of a 59-year-old woman with community-acquired *Roseomonas mucosa* sepsis who presented with persistent fever progressing to septic shock, despite repeatedly negative host-response biomarker results. Initial metagenomic analysis of peripheral blood suggested *Pseudomonas aeruginosa* infection. However, a peripheral blood culture identified *Roseomonas mucosa* as the causative pathogen. She was cured after switching to meropenem according to blood cultures and antimicrobial susceptibility testing.

## Introduction

Species of the genus *Roseomonas* have been reported to cause significant infections in immunocompromised patients, with bloodstream infections being the most common clinical manifestation (1–3). Due to their inherent resistance to β-lactams (including penicillin, piperacillin/tazobactam, and cephalosporins) and variable resistance to quinolones, early identification and targeted antimicrobial therapy are critical for improving clinical outcomes (3–5).

Sepsis, a life-threatening organ dysfunction caused by dysregulated host response to infection, remains a leading global cause of mortality (6). This clinical syndrome is distinguished from uncomplicated bloodstream infection by the presence of systemic inflammatory response accompanied by acute organ failure (7). Inflammatory biomarkers are commonly used to differentiate between distinct pathogenic conditions, indicate disease severity, guide treatment approaches, monitor therapeutic responses, and predict patient prognoses (8). The most common biomarkers of acute inflammation include procalcitonin (PCT), C-reactive protein (CRP), and erythrocyte sedimentation rate (ESR)—which are all typically elevated during inflammation and gradually decline after it is controlled (9).

Herein, we report a rare case of community-acquired *Roseomonas mucosa* (*R. mucosa*) sepsis where the patient consistently presented with normal PCT, CRP, and ESR levels. Despite peripheral blood metagenomic next-generation sequencing (mNGS) results suggesting that the infection was caused by *Pseudomonas aeruginosa*, a piperacillin-tazobactam treatment regimen subsequently proved to be ineffective, and the patient ultimately recovered after being switched to meropenem.

## Case presentation

A 59-year-old woman was admitted to the infectious diseases ward of our hospital with recurrent fever lasting 4 days, wherein her highest body temperature reached 39.9°C. Prior to hospitalization, she developed fever, headaches, and fatigue. She had taken oral cefdinir for 2 days, but it proved ineffective. A post-admission physical examination revealed no significant abnormalities in the heart, lungs, or abdomen. She had been living with type 2 diabetes mellitus for 10 years, and had no prior history of hospitalization before the onset of her illness. She had not received any immunosuppressive therapy. She had been living in the city, had not traveled recently, and had no relevant history of contact with animals. Laboratory tests showed that her white blood cell (WBC), PCT, CRP, and ESR counts were all within normal range. Tests for respiratory adenovirus, rhinovirus, influenza A virus, influenza B virus, SARS-CoV-2, cytomegalovirus, rubella virus, Epstein-Barr virus, mycoplasma, and chlamydia were all negative, as were β-D-glucan (G) and galactomannan (GM) assays. Computed tomography (CT) did not reveal any signs of acute infection, and a cardiac ultrasound showed normal results. We administered piperacillin-tazobactam as an empirical treatment for bacteria on the first day of the patient’s hospitalization; however, she continued to experience recurrent high fever accompanied by chills and hypotension, with a Sequential Organ Failure Assessment (SOFA) score of 3. Despite this, her inflammatory markers (WBC, CRP, PCT, ESR, and interleukin 6) were within normal ranges, and blood cultures remained negative for 3 days.

**Figure 1:**
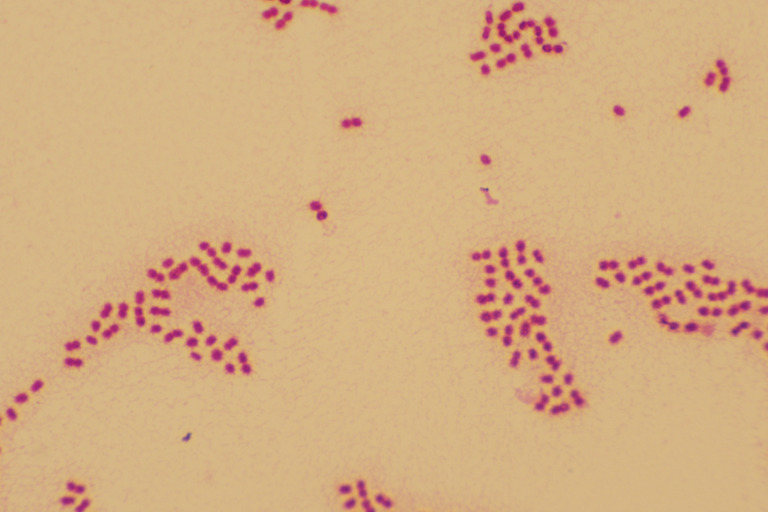
Gram stain (×1000), Roseomonas mucosa is a pink-pigmented, Gram-negative short rod bacterium.

**Figure 2:**
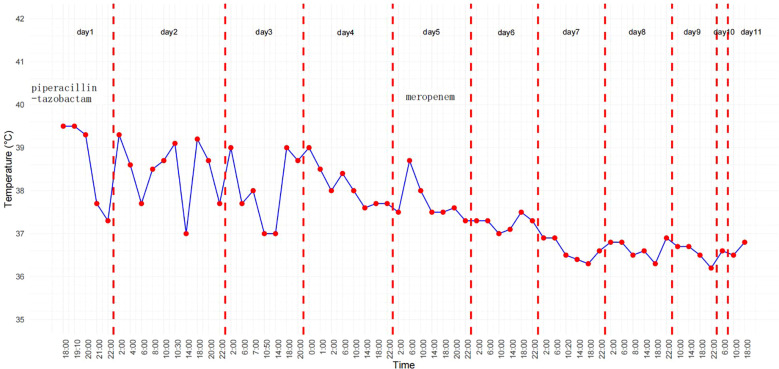
Hospitalization temperature curve.

On the fourth day following her admission, we performed a peripheral blood mNGS test, which detected *P. aeruginosa* with 381 sequence reads. We considered the possibility that the antibiotic therapy regimen already administered had been insufficient, so we decided to continue it. However, the patient remained febrile, with an unchanged peak fever temperature, and displayed early signs of developing shock, as evidenced by a blood pressure drop to 84/42 mmHg. Therefore, on the fifth day, we changed her antibiotic regimen to meropenem. On the seventh day, blood culture test results suggested that the pathogen responsible for her infection was actually *R. mucosa* (**Figure 1**). This organism is resistant to piperacillin-tazobactam but sensitive to meropenem. The patient’s temperature began to decrease and her blood pressure returned to normal after her treatment regimen was switched. By the eighth day, her temperature had also normalized. After receiving meropenem for seven days, additional blood culture test results were also negative, indicating that the infection had been controlled. The temperature graph in **Figure 2** visually presents the fever pattern of the patient throughout the treatment period. She was subsequently discharged from the hospital and followed up for 6 months, during which there was no recurrence of fever.

## Discussion and conclusion

Infections caused by *Roseomonas* spp. in humans are rare. However, they can infect immunocompromised patients, such as those with leukemia undergoing chemotherapy, active malignancies, peritonitis, diabetes, catheter-associated tuberculosis or pulmonary tuberculosis (2, 5, 10). In this case report, we describe a patient who suffered from septic shock caused by *R. mucosa*, but exhibited persistently normal host-response biomarkers, including PCT, CRP, and ESR, throughout the illness. This severe infection showed different results between mNGS tests and blood cultures. The patient was successfully treated with meropenem after undergoing antimicrobial susceptibility testing.

Species of the *Roseomonas* genus belong to the phylum *Proteobacteria*. They are pink-pigmented, slow-growing, non-fermentative, aerobic gram-negative coccobacilli, with a mortality rate of 3% in infected adult patients (11). Patients typically present with fever in 62.5% (45/72) of cases, and with sepsis in 27.8% (10/36) of cases (3). In general, *R. mucosa* is resistant to β-lactam antibiotics such as piperacillin-tazobactam, ampicillin, extended-spectrum cephalosporins, and colistin, but is fully susceptible to aminoglycosides, fluoroquinolones, and carbapenems (12, 13). Our patient presented with fever and septic shock, and antimicrobial susceptibility testing suggested significant resistance to piperacillin-tazobactam, intermediate resistance to levofloxacin, and high sensitivity to meropenem. In this case, *R. mucosa* caused sepsis in a patient with diabetes mellitus. We initially suspected that the source of infection was *R. mucosa* due to the presence of an itchy scratch on the patient’s skin one month prior to her admission to hospital. A small skin cut allowed the opportunistic pathogen to enter the immunocompromised host, ultimately causing sepsis. Immunocompromised patients with bloodborne infections should therefore always be thoroughly evaluated for history of skin damage. Early and accurate identification of the causative pathogen is crucial for effective treatment.

Sepsis is often accompanied by elevated levels of systemic inflammatory markers such as PCT and CRP (9, 14). Both of these markers are associated with a systemic host response (15). A systematic review and meta-analysis updated in 2018, including nine studies comparing the diagnostic accuracy of PCT vs CRP for sepsis, revealed a similar sensitivity for these two biomarkers (CRP: 0.80, 95% confidence interval [CI]: 0.63–0.90, PCT: 0.80, 95% CI: 0.69–0.87) but significantly lower specificity for CRP, at 0.61 (95% CI: 0.50–0.72), compared to PCT, at 0.77 (95% CI: 0.60–0.88) (16, 17). It is important to note that sepsis is a complex and heterogeneous condition. Patients may exhibit varying immune responses to the condition, leading to different levels of biomarker expression. Factors such as age, organ dysfunction, infection type, and comorbidities can all influence biomarker profiles. Dysregulated immune response in sepsis varies significantly among individuals. Some patients may not mount a robust inflammatory reaction, resulting in minimal elevation of typical biomarkers (18). As in this case, multiple negative tests for PCT and CRP did not suggest that the patient’s inflammatory response was mild or that the infection was under control. Conversely, the patient presented with septic shock, indicating severe infection. This may have been because the patient had been suffering from diabetes mellitus and associated chronic hyperglycemia—a complex clinical syndrome often accompanied by immunomodulatory dysfunction—for many years (19). Studies have found that long-term hyperglycemia can continuously activate the NF-kappaB pathway through oxidative stress, leading to elevated baseline levels of pro-inflammatory factors such as IL-6, which may blunt the magnitude of inflammatory response during acute infection (20). In the study by Al-Rashed F et al., it was also shown that monocytes from diabetic patients had impaired TLR4 signaling and reduced TNF-α release upon LPS stimulation, resulting in insufficient CRP synthesis (21).Another contributing factor may be the patient’s advanced age and immunosenescence, combined with delayed effective antimicrobial therapy during early infection, potentially leading to pathogen dissemination and progression to sepsis. Therefore, the diagnosis of sepsis should rely on a combination of clinical criteria, biomarkers, and imaging. The absence of specific biomarker elevation does not rule out sepsis, particularly in cases with atypical presentations or those in their early stages (22).

Recently, mNGS has emerged as a revolutionary tool for its use in pathogen detection. Compared to traditional blood cultures, mNGS is faster and more sensitive in pathogen detection (23, 24). However, owing to host DNA interference and database heterogeneity, mNGS may not be as effective as blood culture in terms of its specificity for identifying the causative pathogen. In our case, an mNGS test of the patient’s peripheral blood detected *P. aeruginosa*, whereas blood cultures detected *R. mucosa*. Based on the therapeutic failure of piperacillin-tazobactam treatment, we considered the actual etiological pathogen to be *R. mucosa*.

To the best of our knowledge, this is the first report in the literature of sepsis caused by *R. mucos*a with normal host response inflammatory biomarkers. In this case, blood cultures proved to be more accurate than mNGS. Therefore, it is crucial to thoroughly analyze mNGS reports in the context of each patient’s actual clinical condition.

Sepsis caused by *R. mucosa* is relatively rare, difficult to diagnose, and may progress rapidly. To improve patient prognoses, clinicians require use multiple tools to clarify its etiology as soon as possible, and appropriate antimicrobials must be selected rationally, with adequate screening for multiple potential pathogens.

## Data Availability

The original contributions presented in the study are included in the article/supplementary material. Further inquiries can be directed to the corresponding author.
